# Brain activation patterns in medicated versus medication-naïve adults with attention-deficit hyperactivity disorder during fMRI tasks of motor inhibition and cognitive switching

**DOI:** 10.1186/s12880-021-00579-3

**Published:** 2021-03-19

**Authors:** Jatta Berberat, Ruth Huggenberger, Margherita Montali, Philipp Gruber, Achmed Pircher, Karl-Olof Lövblad, Hanspeter E. Killer, Luca Remonda

**Affiliations:** 1 Department of Neuroradiology, Kantonsspital Aarau, Tellstrasse 25, 5001 Aarau, Switzerland; 2 Praxisgemeinschaft Theaterplatz, 5400 Baden, Switzerland; 3 Department of Ophthalmology, Kantonsspital Aarau, 5001 Aarau, Switzerland; 4 Department of Radiology and Medical Informatics, University of Geneva, 1202 Geneva, Switzerland; 5 University of Bern, 3011 Bern, Switzerland

**Keywords:** ADHD, Adults, Functional MRI, Neuroimaging, Diagnosis

## Abstract

**Background:**

Adult-attention-deficit-hyperactive-disorder (ADHD) is often unrecognized condition. FMRI examination along with neuropsychological testing might strengthen the diagnosis. We hypothesized that ADHD-adults with and without medication would show different fMRI pattern compared to healthy controls while testing tasks of motor inhibition and cognitive switching.

**Methods:**

45 subjects in three age-matched groups: (1) controls, (2) ADHD-adults under medication (ADHD+) and (3) medication-naïve adults with ADHD (ADHD−) underwent fMRI and neuropsychological testing. Group analysis and population-based statistics were performed.

**Results:**

DTVP-A, intellectual ability as well as attention capability, visual-perceptual and visual-motor abilities showed no significant differences between the groups. However, fMRI revealed statistically significant differences between the ADHD+, ADHD− and control groups on tasks of motor inhibition and cognitive switching on adults in bilateral fronto-striatal brain regions, inferior fronto-frontal, fronto-cingulate and fronto-parietal networks as well as in the parietal lobe (*p* < 0.05).

**Conclusions:**

fMRI offers the potential to differentiate between the ADHD+, ADHD− and control groups. FMRI possibly opens a new window for monitoring the therapeutic effect of ADHD medication.

**Trial registration:**

NCT02578342, registered at August 2015 to clinical trial registry (https://ichgcp.net/clinical-trials-registry/NCT02578342).

## Background

Adult Attention Deficit Hyperactivity Disorder (ADHD) is a relatively common, but often unrecognized condition. ADHD is characterized by age-inappropriate symptoms of inattention, impulsiveness and hyperactivity [27]. It affects academic and social development and is associated with significant psychiatric comorbidities and mental health problems in adult life [3, 40]. Most of these adults live with the symptoms and suffer the often devastating effects of ADHD in their lives without identifying the source of their struggles. Underlying symptoms can be hidden under depression, anxiety, burn out, border line personality, bipolar disorder and substance abuse [25].

The most consistent neuropsychological findings in adults with ADHD are deficits in motor response inhibition and cognitive switching [5, 10]. Recent meta-analyses of task-based on functional MRI (fMRI) studies show domain-specific brain dysfunctions in ADHD based on motor response (Go/No-Go task, Stop task and Switch task) bilateral frontal- and frontoparietal areas, occipital, parietal and temporal areas as well as insula when compared to controls [8, 18, 28, 30, 35].

Three modified tasks of inhibitory control that have earlier been described in fMRI studies in adults with ADHD [10, 25]: Go/No-Go and Stop tasks were used to study the response inhibition, whereas a visual-spatial Switch task was used to require the inhibition of previously valid stimulus–response associations. Go/No-Go and Stop tasks have been used to show reduced activation in inferior prefrontal cortex, caudate and anterior cingulate [25, 39] and Switch task has shown to elicit reduced activation in inferior prefrontal cortex, striatum and parietal regions [1, 25] on ADHD patients.

Stimulant medication history is a major confounder in imaging studies, given evidence for long-term effects of stimulant medication on brain structure [23] and brain function [33]. Therefore, we studied 3 groups of age-matched participants: healthy controls, medication-naïve (ADHD−) and medicated adults with the diagnosis of ADHD (ADHD+).

We hypothesized that inattentive/hyperactive adults with ADHD have different activation patterns in fronto-striatal and fronto-parietal brain regions compared to healthy comparison adults.

## Methods

### Subjects

All participants were over 20-year old adults. They were divided into three groups: controls (n = 15), subjects with ADHD under medication (ADHD+, n = 15) and medication-naïve ADHD subjects (ADHD−, n = 15). All subjects had normal or corrected-to-normal visual acuity. The three groups of the study were age matched as follows: (1) controls (3 males, age 22–55 years old (mean age 38 ± 12 year), all right handed), (2) ADHD+ (3 males, age 21–58 years old (mean age 38 ± 8 year), all right handed) and (3) ADHD− (8 males, age 29–56 years old (mean age 43 ± 8 year), 2 left handed).

The study was performed in agreement with the Declaration of Helsinki. All subjects gave written informed consent and the study was approved by the local ethical committee. Exclusion criteria for controls were present or past history of any mental disorder and substance abuse. Since the stimulant medication history is a major confounder in imaging studies [10], all ADHD participants under medication were only included in the study if they had achieved a stable phase with the medication doses (min. 6 months under medication [Concerta: methylphenidate hydrochloride; Janssen-Cilag AG, Schaffhausen, Switzerland)]. Exclusion criteria for subjects with ADHD were all neurological diseases that could interfere with a diagnosis of ADHD, psychiatric comorbidity under treatment, previous history of psychiatric medication or substance abuse; among them depression, dysthymic disorders, posttraumatic stress disorder and anxiety disorder.

Intellectual ability (IQ) was assessed with the German version of the KAIT (K-TIM; Testzentrale den Schweizer Psychologen AG, Bern, Switzerland). Attention capability, visual-perceptual and visual-motor abilities were assessed by the d2 test and the Developmental Test of Visual Perception–Adolescent and Adult (DTVP-A, PRO-ED, Inc., Texas, US), respectively.

### MRI examination

All subjects underwent fMRI at 3T (Skyra, Siemens Healthcare, Erlangen, Germany) with a 32-channel head coil. FMRI was performed utilizing an echo planar imaging sequence (EPI, slice thickness 3 mm, repetition time (TR) = 2000 ms, echo time (TE) = 30 ms, flip angle 90°). T1-weighted anatomical, 3-dimensional, volumetric, interpolated brain examination sequence (fat saturated, TR = 1940 ms, TE = 367 ms, TI = 900 ms, voxel size 1 × 1 × 1 mm, flip angle 9°, 192 slices, acquisition time (ta) = 4.3 min) with T2-weighted turbo spin echo (tse, TR = 6250 ms, TE = 100 ms, voxel size 0.4 × 0.4 × 4 mm, flip angle 135°, 30 slices, ta = 2.3 min) followed.

### Experimental design

fMRI tasks were built in as block-paradigms and all stimuli were presented in the center of the screen. The visual stimuli were programmed using E-Prime software (Psychology Software Tools, Sharpsburg, PA, USA). Subjects laid in the scanner and viewed the stimuli displayed on a monitor via a mirror (NordicNeuroLab SA, Bergen, Norway). Participants practiced the first trial block per task to familiarize themselves with the test.

In the “*Go/No-Go task”*, No-Go signals are presented intermixed with Go-signals [25], calling for action restraint [15, 19, 20]. At the Go/No-Go task [2, 32, 37], letters X (right button press) and O (left button press) were used as Go-signal, letters “K, L, M, N” as No-Go signal. Randomized signal changed every two seconds. Reaction times, error rates and percentage of missing Go-trials were computed. Four alternating blocks were presented, each comprising 16 trials. Between the Go-trials, a baseline block was presented as crosshair (duration of eight signals of 2000 ms each, total of 4 blocks). Task duration was 4.6 min.

In “*Stop task”* similar to Rubia et al. [1, 22] arrows of 2000 ms duration each were randomly pointed either to the left, right, up or down in the middle of the screen with a stimulus interval of 2000 ms. Subjects were instructed to perform a button response with their dominant hand thumb corresponding to the direction of the arrow. In the unpredictable, infrequent Stop-trials (20% of trials) subjects had to inhibit their motor response while the arrows pointing left and right or up and down at the same time. Between the Stop-trials a baseline blocks were presented as a crosshair, duration of eight signals each. The experiment included six Stop-task blocks (18 trials, duration 2 s each), in between six baseline blocks (8 trials, duration 2 s each). Task lasted total 4.7 min.

The “*Switch task*” requires cognitive switching between two spatial dimensions [3, 11, 14]. It was built as a task repetition, in which the current cue was different from the previous cue but specified by the same task. A target dot appearing in one of the four corners of a grid with an arrow in the middle. The subject had to decide whether the target is on the left or right or whether is in the superior or inferior part of the grid depending on the direction of the arrow (respectively vertical or horizontal). Situation on the task changed every 2000 ms. Four alternating blocks were presented, each comprising fifteen signals (duration 2000 ms each). Between the Go-trials a baseline blocks were presented as crosshair, duration of eight signals, 2000 ms each. Total task duration was 3.4 min.

### Data analysis

#### fMRI

Data was analyzed individually for all three groups. Blood oxygen level dependent (BOLD) signal changes were analyzed and resulting contrast maps were created for a response to cognitive tasks versus crosshair. BOLD clusters were assessed individually with respect to the anatomical location. Data was analyzed using Brainvoyager QX software (Brain Innovation, Maastricht, The Netherlands) consisting of the following steps: motion correction (trilinear or sync interpolation by spatial alignment for all acquired volumes by rigid body transformation), co-registration of functional imaging and 3-dimensional isovoxel anatomic data and spatial smoothing (Gaussian filter of 4 mm full width half maximum (FWHM)). Anatomical images were then transferred to the Talairach space. At the first level of analysis, a general linear model was computed for each experiment, applying separate predictors for each subject. Then, multi-subject analysis was applied by averaging all the data. Activation maps were corrected for multiple comparisons using false discovery rate (FDR) approach with *p* < 0.05, considering a minimum cluster of more than 20 contiguous voxels in terms of t-statistics based on BOLD signal changes.

For population-based statistics, random-effect (RFX) analysis was performed that takes into account the between- and intra-subject variability. The different subjects are treated as random samples from the possible selection of subjects. The RFX analysis calculates beta (β) weights per subject and predictor. The resulting BOLD activation map shows a comparison of the individual betas of all subjects. In this way, the variability between the different subjects can be calculated and, thus, conclusions can also be drawn for the general population. Gaussian smoothing of FWHM of 4 mm, and min. cluster of 20 voxels was used.

Groups were compared by computing the mean of the summary statistic (contrast) for each group followed by a false discovery rate (FDR) corrected t-statistics (FDR < 0.05). Using FDR correction one is able to control the number of false positive voxels among the subset of voxels labeled as significant on multiple comparisons.

#### Structural MRI

Clinical MR images were viewed by two board certified neuroradiologists.

#### Statistics

Descriptive statistics were used to characterize the patient population. Data are presented as mean ± standard deviation (SD). To determine whether there are statistically significant differences of the behavioural data between the three groups (controls, ADHD+ and ADHD−) one-way analysis of variance ANOVA. Correlation was calculated using Sperson's linear Correlation. All statistics were performed using SPSS (version 24, IBM, New York, USA). Outliers were defined, in case the results would have been outside the group’s mean ± 2SD.

## Results

### Neuropsychological tests

DTVP-A, intellectual ability as well as attention capability, visual-perceptual and visual-motor abilities tested in the d2 test revealed no statistically significant differences between the three groups. Results are summarized in Table 1.

**Table 1:** Summary of subject information

	Controls (n = 15)	ADHD+ (n = 15)	ADHD− (n = 15)
*Neuropsychology*			
K-TIM test 9 (%)	110 ± 16 (100–137)	128 ± 15 (98–140)	118 ± 12 (96–135)
DTVP-A (%)	59 ± 20 (25–91)	47 ± 25 (16–84)	47 ± 23 (8–92)
*Response time on fMRI (s)*			
Response time	0.727 ± 0.096 (0.632–0.865)	0.666 ± 0.130 (0.553–1.055)	0.718 ± 0.160 (0.596–0.814)
Correct answers (%)	100 ± 1 (97–100)	96 ± 6 (85–100)	100 ± 0 (100)

Results are presented as mean ± standard deviation. Range is presented in the brackets

*R* ight, *L* left

Since the stimulant medication history is a major confounder in imaging studies, all ADHD participants under medication were studied after they reached a steady state with the medication doses (min. 6 months after starting the medication). For the rest of the ADHD+ group the imaging examination was done after 1–11 years (mean 3.8 ± 3.3 year) since they got their diagnosis and started the medication. The ADHD− group had 8 newly diagnosed adults, the rest ranged between 1 to 22 years (mean 5.3 ± 7.0 year).

### fMRI

The average response time is summarized in Table 1. The rate of the correct answers was the same for all three groups (99.8% ± 0.77%). in the speed of response inhibiting, two persons from the ADHD+ group were outliners in their long response time. However, ADHD+, ADHD− and controls revealed no statistically significant differences when compared to each other. The maximum absolute displacement derived from the fMRI data was similar to each group varying between 0.5 to 3 mm, was successfully corrected by the data post processing and no statistically significant differences were detected between the ADHD+, ADHD− and healthy volunteers, respectively.

When correlating IQ, DTVP-A and motion displacement within the groups, no statistically significant correlations were found.

A list of activated brain regions of interest with respective *t*-values are listed in Table 2 for all the groups during the “Go/No-Go task”, Table 3 summarizes the results of the “Stop task” and Table 4 extends the results to the “Switch task”. The results of the group comparison are presented in Table 5.

**Table 2:** fMRI activation on Go/No-Go task

		Talairach			No. of voxels	t	Talairach			No. of voxels	t	Talairach			No. of voxels	t
		x	y	z			x	y	z			x	y	z		
		Controls					ADHD+					ADHD−				
Inferior frontal gyrus	R	42	11	4	1238	3.6	46	19	− 1	48	2.5	42	11	4	2367	5.3
	L	− 46	9	4	383	4.8	− 46	15	0	531	3.9	− 46	4	4	1341	6.4
Insula	R	42	11	4	1067	3.9	–	–	–	–	–	42	11	6	2015	4.6
	L	− 46	12	4	313	4.8	− 47	16	0	100	3.4	− 42	12	4	1188	4.3
Postcentral gyrus/insula	R	53	− 6	11	2525	4.5	–	–	–	–	–	53	− 2	11	2383	5.2
	L	− 58	− 5	11	654	4.8	–	–	–	–	–	− 54	− 1	11	1345	5.2
Putamen	R	21	3	0	183	3.4	16	8	− 6	84	2.7	21	2	1	1005	5.1
	L	− 26	10	7	63	3.1	− 22	10	0	13	2.3	− 21	7	5	552	4.8
Caudate	R	14	10	16	24	2.4	–	–	–	–	–	14	13	16	546	3.6
	L	− 14	10	16	26	3.0	− 22	10	16	6	2.3	− 18	13	16	462	4.3
Thalamus	R	14	− 25	3	105	2.9	14	− 29	− 3	42	2.6	27	− 19	3	39	2.8
	L	− 7	− 25	9	194	4.9	− 15	− 30	− 2	152	4.4	− 16	− 17	8	346	4.6
ACC (/caudate/thalamus)	R	15	− 7	27	27	2.5	–	−	−	−	−	13	24	22	2165	5.1
	L	− 5	12	21	214	4.0	–	–	–	–	–	− 13	28	17	1184	4.3
Pre-SMA		0	− 6	46	759	4.0	− 4	6	46	55	2.6	2	6	46	406	4.6
Precuneus	R	9	− 62	50	748	4.2	–	–	–	–	–	9	− 58	52	894	3.7
	L	− 15	− 46	50	873	3.5	− 24	− 66	47	284	3.3	− 8	− 53	51	1722	5.4
Globus pallidus	R	16	− 4	− 8	199	4.0	–	–	–	–	–	18	0	–8	281	4.2
	L	− 9	− 1	− 8	230	2.7	–	–	–	–	–					
Putamen/parahippocampal gyrus/amygdala(/pallidus)	R	23	0	− 8	149	3.7	23	11	− 8	83	2.9	18	− 4	− 16	172	3.6
	L	− 24	0	− 8	6	2.0	–	–	–	–	–	− 17	4	− 8	211	4.2
Superior parietal gyrus(/precuneus)	R	18	− 64	49	1281	4.3	–	–	–	–	–	30	− 57	47	658	3.7
	L	− 33	− 61	49	830	3.6	− 18	− 63	42	104	2.9	− 30	− 57	47	1704	4.2
Inferior parietal gyrus(/orecuneus/superior parietal gyrus)	R	30	− 57	42	29	2.6	41	− 51	42	6	2.5	48	− 50	42	210	5.4
	L	− 42	− 54	42	105	3.4	− 42	− 49	42	45	2.7	− 45	− 50	45	484	3.1
Inferior temporal gyrus/fusiform gyrus/occipital	R	38	− 64	− 11	2364	4.7	38	− 67	− 14	927	4.4	38	− 63	− 13	1557	5.6
	L	− 43	− 63	− 11	2529	4.1	− 36	− 66	− 14	1104	5.3	− 49	− 72	0	1341	3.9

*R* right, *L* left, *ACC* anterior cingulate cortex, *SMA* supplementary motor area

**Table 3:** fMRI activation based on Stop task

		Talairach			No. of voxels	T	Talairach			No. of voxels	T	Talairach			No. of voxels	t
		x	y	z			x	y	z			x	y	z		
		Controls					ADHD+					ADHD−				
R Inferior prefrontal cortex	R	41	25	− 2	2196	4.2	45	24	− 2	2074	5.0	41	26	− 2	1642	6.2
L Inferior prefrontal cortex	L	− 43	25	− 7	1036	4.1	− 43	22	− 3	1045	4.9	− 44	26	− 3	836	3.8
Mesial frontopolar cortex	R	5	41	16	180	2.9	− 1	55	12	99	3.1	− 4	55	12	407	3.3
R Rostral anterior cingulate	R	13	40	13	472	4.6	–	–	–	–	–	1	42	16	496	4.5
L Rostral anterior cingulate	L	− 15	40	9	27	2.5	–	–	–	–	–	− 13	40	16	1103	4.5
R Inferior parietal lobe	R	46	− 49	26	410	3.9	47	− 43	26	209	4.2	51	− 49	28	2190	4.7
L Inferior parietal lobe	L	–	–	–	–	–	− 46	− 36	27	11	2.7	− 64	− 36	27	1744	6.2

*R* right, *L* left,

**Table 4:** fMRI activation on Switch task

		Talairach			No. of voxels	T	Talairach			No. of voxels	T	Talairach			No. of voxels	t
		x	y	z			x	y	z			x	y	z		
		Controls					ADHD+					ADHD−				
Inferior frontal gyrus	R	42	11	4	2520	6.3	45	27	− 8	2982	5.0	42	11	4	2327	5.0
	L	− 46	9	4	1694	5.2	− 43	30	− 13	1748	4.4	− 42	11	4	2107	5.6
Insula	R	42	11	4	1489	5.7	42	15	4	494	5.0	46	12	4	1904	5.0
	L	− 46	12	4	1361	6.9	− 42	13	4	2838	4.7	− 41	18	4	2659	6.1
Caudate	R	–	–	–	–	–	16	3	22	105	4.2	16	8	21	906	4.7
	L	− 13	10	22	109	3.5	− 13	4	19	520	4.9	− 16	6	22	441	3.6
Putamen	R	21	− 3	− 4	47	3.3	15	− 3	− 2	46	2.5	–	–	–	–	–
	L	− 28	11	7	166	5.2	− 16	− 8	0	376	3.9	− 28	2	6	185	5.7
Precentral gyrus	R	44	− 1	52	120	3.7	31	− 4	52	1631	5.6	46	3	52	1626	4.6
	L	− 38	− 1	45	259	4.1	− 56	0	42	2456	6.2	− 46	3	52	362	3.7
Postcentral gyrus	R	57	− 6	12	1275	5.9	57	− 6	12	1504	4.7	57	− 6	12	1601	4.6
	L	− 58	− 5	11	1264	4.2	− 57	− 6	14	1197	4.3	− 62	− 5	12	1417	5.6
Inferior parietal gyrus(/orecuneus/superior parietal gyrus)	R	30	− 58	42	1373	5.3	36	− 57	42	2037	6.8	45	− 58	42	1409	4.1
	L	− 42	− 58	42	633	4.7	− 41	− 57	42	2981	7.8	− 48	− 58	40	148	3.2

*R* right, *L* left,

**Table 5:** Group comparison based on the Random-effect (RFX) analysis (p < 0.05) with FDR correction for multiple comparisons (FDR < 0.05)

		Talairach			Go/No-go task						Stop task						Switch task					
					No. of voxels	t	No. of voxels	t	No. of voxels	t	No. of voxels	t	No. of voxels	t	No. of voxels	t	No. of voxels	t	No. of voxels	t	No. of voxels	t
		x	y	z	Controls versus ADHD+		Controls versus ADHD−		ADHD+ versus ADHD−		Controls versus ADHD+		Controls versus ADHD−		ADHD+ versus ADHD−		Controls versus ADHD+		Controls verss ADHD−		ADHD+ versuss ADHD−	
Inf. prefrontal cortex	R	34	31	10	20	2.2	743	2.7	94	2.6	63	2.8	452	5.0	50	2.4	547	4.8	453	2.6	692	4.4
	L	− 33	44	16	25	2.3	249	4.3	412	3.5	27	2.3	446	4.3	105	3.4	344	4.3	369	4.1	891	4.5
Inferior frontal gyrus	R	42	27	5	23	2.2	378	2.4	270	3.2	121	3.7	922	6.6	76	3.3	847	6.1	941	6.2	504	5.2
	L	− 42	27	5	96	2.5	536	2.8	401	4.9	406	4.3	917	5.2	49	3.0	872	4.8	953	5.0	886	5.3
Insula	R	42	11	4	101	2.4	712	4.9	250	3.8	–	–	591	5.1	407	6.0	605	5.2	534	4.9	648	5.1
	L	− 46	12	4	114	2.3	–	–	–	–	–	–	871	5.8	583	5.6	850	5.6	871	5.8	978	6.7
Putamen	R	21	0	− 3	24	2.1	335	2.6	55	2.9	20	2.3	47	3.6	26	2.8	34	3.2	50	3.6	20	2.4
	L	− 27	1	7	26	2.2	458	2.4	213	3.8	29	2.4	621	4.9	280	3.5	475	4.0	484	4.1	378	4.1
Thalamus	R	14	− 25	3	34	2.3	20	2.2	20	2.4	122	3.2	292	4.3	243	4.6	201	4.1	20	2.3	150	3.4
	L	− 7	− 25	9	45	2.4	136	4.5	111	2.9	163	3.2	90	3.3	273	3.9	50	3.0	90	3.3	322	5.3
Caudate	R	11	0	18	24	2.0	271	3.6	27	2.3	62	2.9	20	2.7	168	3.9	307	4.5	20	2.2	241	3.6
	L	− 14	10	16	–	–	–	–	–	–	20	2.2	110	2.5	263	4.4	247	3.6	224	3.8	318	3.5
ACC	R	15	1	24	20	2.1	261	2.3	89	2.8	131	3.1	275	3.7	20	2.1	135	2.8	164	2.2	273	3.0
	L	− 15	− 7	27	–	––	–	–	–	–	50	3.4	247	2.6	30	3.6	181	3.8	186	3.8	405	4.1
Precentral gyrus	R	47	− 9	50	23	2.3	305	2.8	87	3.4	–	–	–	–	–	–	28	3.0	30	3.0	529	4.8
	L	− 38	− 1	45	–	–	–	–	–	–	–	–	–	–	–	–	116	3.4	292	3.6	675	5.1
Postcentral gyrus	R	50	− 22	47	27	2.2	476	3.4	36	2.5	–	–	–	–	–	–	–	–	–	–	–	–
Precuneus	R	9	− 62	50	–	–	176	3.4	44	3.1	–	–	–	–	–	–	20	2.1	20	2.2	–	–
	L	− 15	− 46	50	–	–	–	–	–	–	–	–	–	–	–	–	121	3.9	111	2.5	263	2.7
Superior parietal gyrus	R	18	− 64	49	–	–	339	4.1	160	2.5	–	–	–	–	–	–	92	3.4	123	3.5	275	4.3
	L	− 33	− 61	49	–	–	–	–	–	–	–	–	–	–	–	–	20	2.3	55	2.7	320	3.5
Inferior parietal gyrus	R	30	− 57	42	–	–	270	4.6	20	2.2	–	–	–	–	–	–	389	4.9	398	5.2	320	3.5
	L	− 42	− 54	42	–	–	–	–	–	–	–	–	–	–	–	–	121	3.2	110	3.1	800	6.6

*R* right, *L* left, *ACC* anterior cingulate cortex

#### Go/No-Go task

**Fig. 1:**
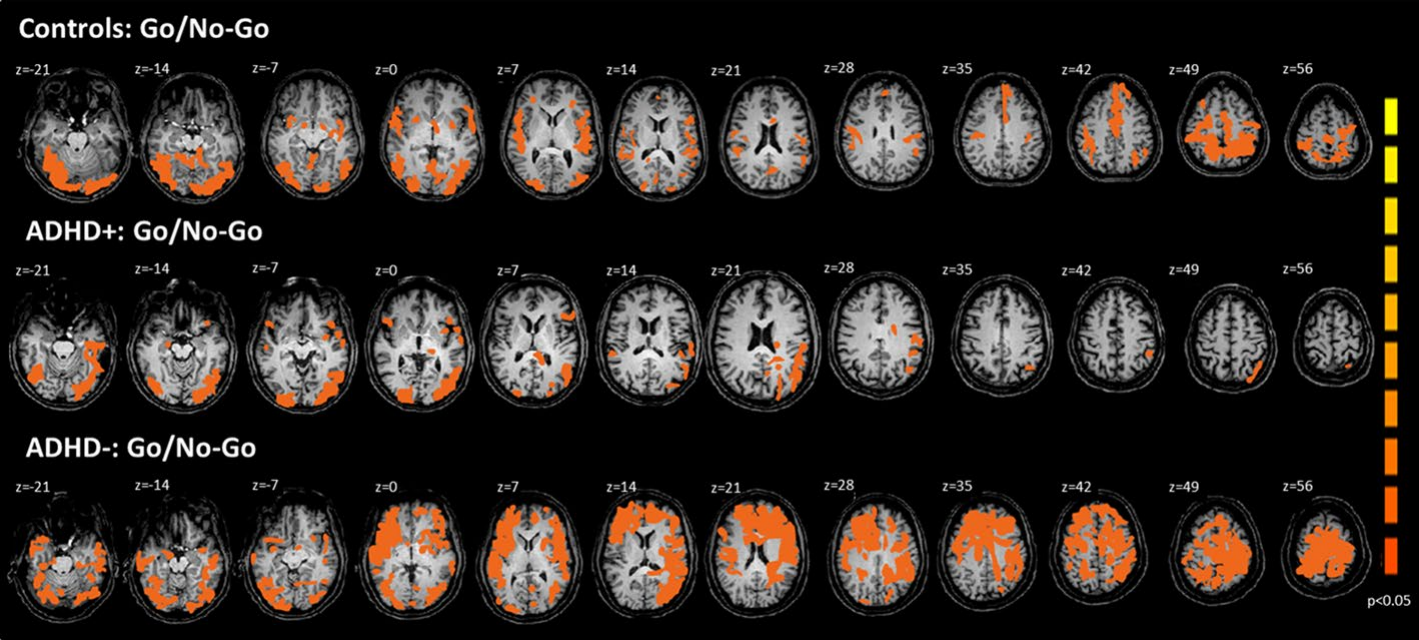
fMRI scan showing statistically significant activation (*p* < 0.05) over the whole brain by pooled data of controls, ADHD+ and ADHD− groups on Go/No-Go task

In the Go/No-Go task ADHD+ group showed diminished activation and ADHD− group increased activation in the following anatomical regions: bilateral on inferior frontal gyrus, insula, postcentral gyrus, putamen, caudate, thalamus, anterior cingulate cortex (ACC), precuneus, globus ballidus and inferior temporal gyrus (Fig. 1, Table 2).

#### 1.1.1. Stop task

**Fig. 2:**
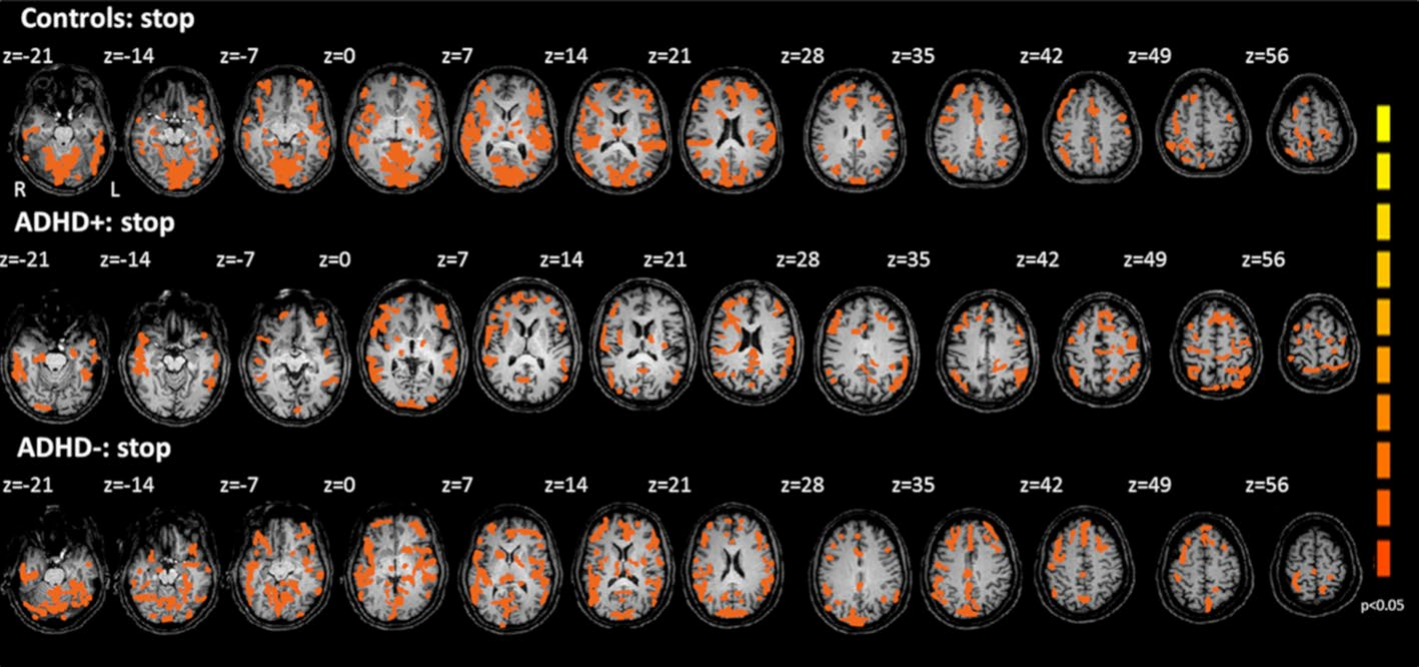
fMRI scan showing statistically significant activation (*p* < 0.05) over the whole brain by pooled data of controls, ADHD+ and ADHD− groups on Stop task

The Stop task revealed differences on the brain activation pattern of the studies groups in inferior prefrontal cortex, inferior frontal gyrus, insula, caudate, putamen, thalamus and ACC (Fig. 2, Table 3). At Stop task ADHD+ group showed no activation on right or left rostral anterior cingulate, whereas controls and ADHD− group showed bilateral rostral anterior cingulate activation. ADHD− group showed hyperactivation on left rostral ACC and bilateral at inferior parietal lobe. By controls no activation was detected at left inferior parietal lobe.

#### Switch task

**Fig. 3:**
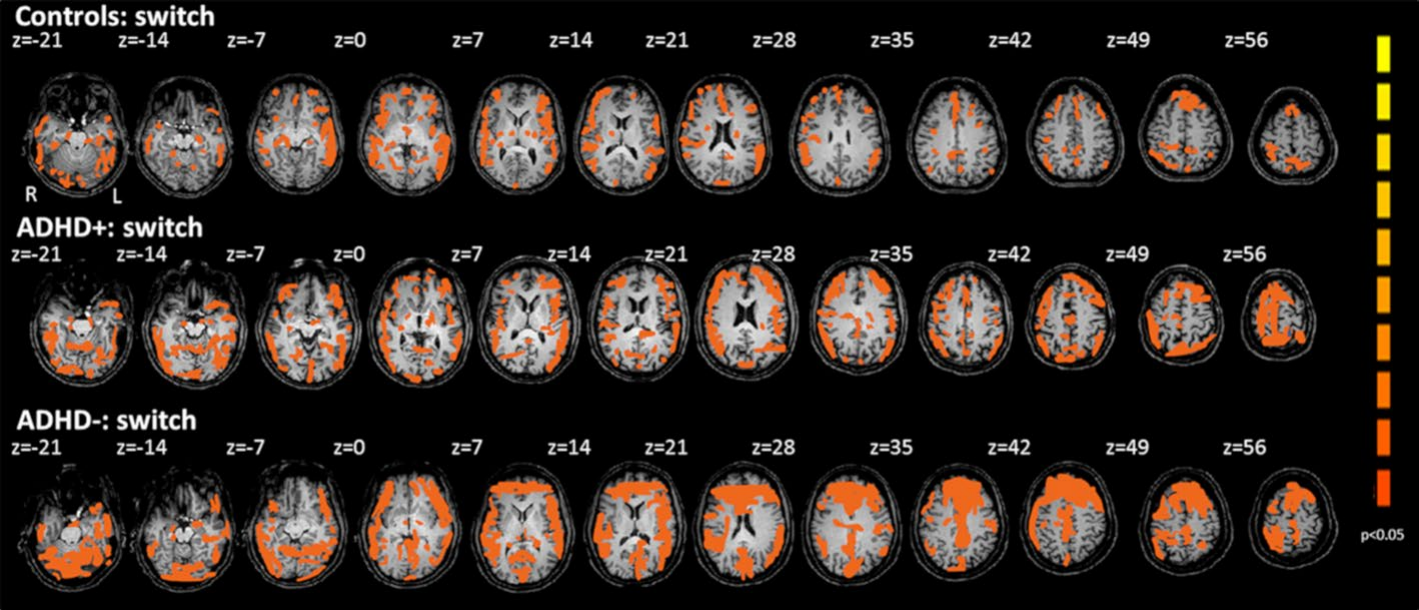
fMRI scan showing statistically significant activation (*p* < 0.05) over the whole brain by pooled data of controls, ADHD+ and ADHD− groups on Switch task

In Switch task ADHD− group revealed bilaterally increased activation on inferior frontal gyrus, insula, caudate, putamen, precentral gyrus and postcentral gyrus (Fig. 3, Table 4). ADHD  group showed hyperactivation on switch task at left insula and bilateral at caudate and putamen as well as bilateral inferior parietal gyrus, precuneus and superior parietal gyrus. ADHD− group showed hyperactivation on the right caudate which was not been on controls or the ADHD+ group, in left inferior frontal gyrus, insula and caudate as well as right precentral gyrus and at the right caudate. ADHD− group showed diminished activation at left inferior parietal gyrus, precuneus and superior parietal gyrus which was not seen by controls or at ADHD+ group.

#### Group comparison

**Fig. 4:**
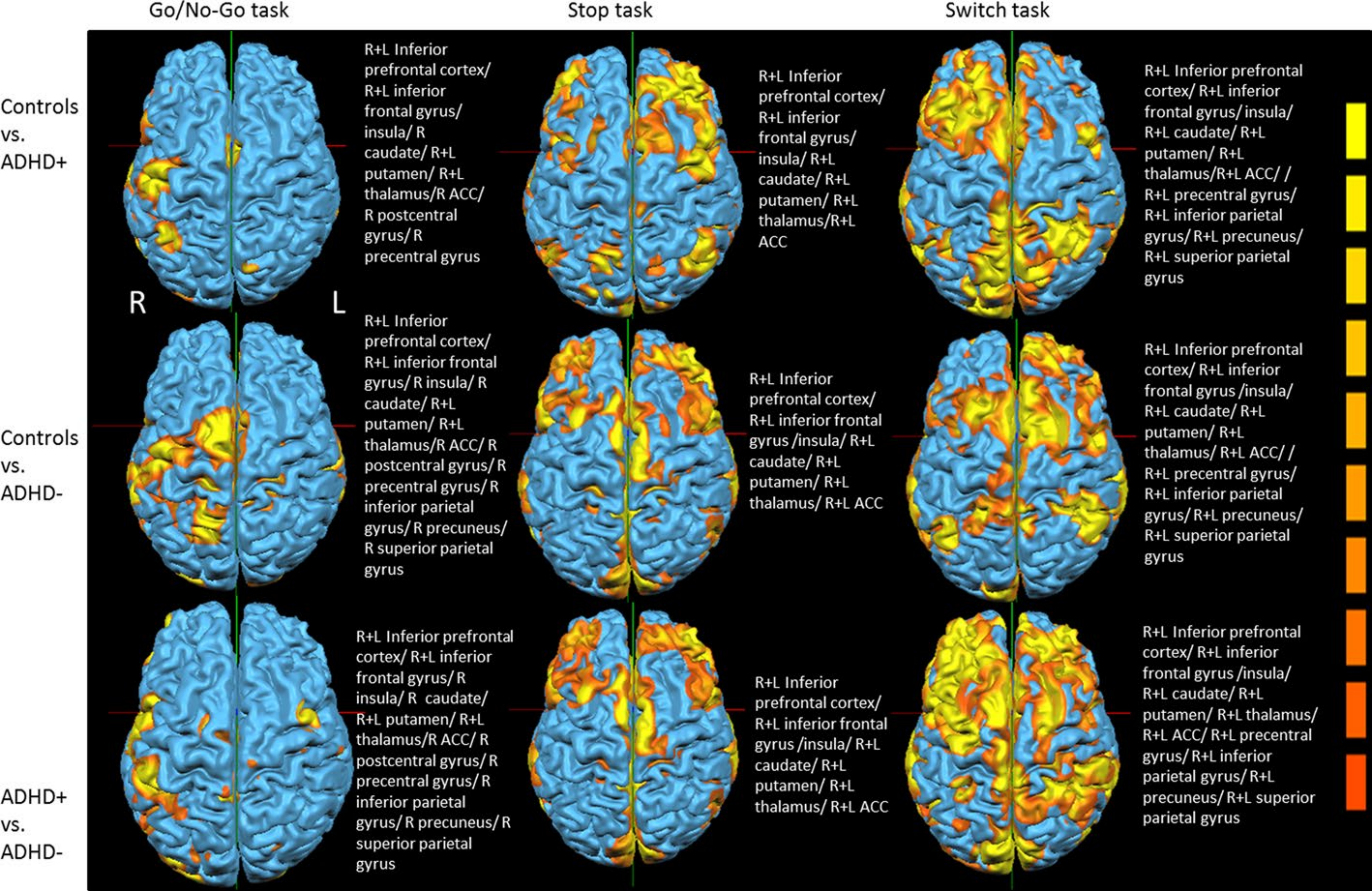
Group comparison based on RFX analysis on Go/No-Go task, Stop task and Switch task

The significant regions of BOLD contrast map of the population effect based on RFX analysis during the tasks of motor inhibition and cognitive switching are presented on Fig. 4 and Table 5: at Go/No-Go task comparison between controls and ADHD+ activation bilateral of inferior prefrontal cortex, inferior frontal gyrus, insula, putamen and thalamus as well as at right caudate, right ACC, right pre- and postcentral gyrus was detected. Comparison between controls and ADHD− as well as ADHD+ versus ADHD− revealed same activation pattern including the following anatomical regions: bilateral of inferior prefrontal cortex, inferior frontal gyrus, putamen, thalamus as well as right caudate, insula, ACC, pre- and postcentral gyrus, inferior parietal gyrus, precuneus and superior parietal gyrus.

When comparing Stop task activation between (1) controls and ADHD+, (2) controls vs ADHD− and (3) ADHD+ versus ADHD− groups, similar activation pattern was revealed in bilateral of inferior prefrontal cortex, inferior frontal gyrus, caudate, putamen, thalamus and ACC. Additionally, when comparing controls vs ADHD− and ADHD+ versus ADHD−, additional insula activation was shown.

At Switch task comparison between (1) controls and ADHD+; (2) controls and ADHD− and (3) ADHD+ and ADHD−; bilateral activation of inferior prefrontal cortex, inferior frontal gyrus, insula, caudate, putamen, thalamus, ACC, precentral gyrus, inferior parietal gyrus, precuneus and superior parietal gyrus was detected. Additionally, between controls versus ADHD+ and controls vs ADHD− bilateral precuneus activation was detected, respectively. ADHD− group revealed hyperactivation pattern compared to the two other groups.

## Discussion

This fMRI study demonstrated a statistically significant differences in the brain activation patterns during motor task inhibition and cognitive switching in adults with and without ADHD, as well as patients with ADHD under and without stimulant medication. These differences in activation regions of BOLD contrast maps based on the group comparison analysis were found at inferior prefrontal cortex, inferior frontal gyrus, insula, putamen and thalamus as well as at right caudate, right ACC, right pre- and postcentral gyrus. These brain regions are responsible in context of ADHD for cognitive skills such as attention, learning, regulating emotions, planning and executing motoric tasks as well as processing the information at working memory.

Dopamine and noradrenaline play important roles in high-level executive functions in attention-deficit/hyperactivity disorder (ADHD) by influencing the fronto-striato-cerebellar circuits [6]. ADHD is associated with reduced noradrenaline (norepinephrine) transporter availability in right attention networks [38]: noradrenaline (norepinephrine) reuptake inhibitors inhibit the uptake of primarily norepinephrine by presynaptic nerve terminals and increase its availability in the synaptic cleft by blocking the human norepinephrine transporter [17]. Dopamine influences the attention, concentration and motivation. We can see this impact of the medication on fronto-striato-cerebellar circuits directly on our fMRI results on both, medicated and medication-naïve ADHD groups compared to controls.

Inhibitory control of behavior, composed of motor, emotional, cognitive, and social acts, as well as error detection are among the highest evolved human self-monitoring functions. Ontogenetically, motor response inhibition has been shown to develop progressively from childhood to adulthood [14]. Inhibiting a motor response that is no longer required is an important aspect of cognitive control. Prefrontal cortex (PFC) in inhibitory motor control is supported by several authors [2, 9, 13]. The inferior, mesial, and dorsolateral prefrontal cortices as well as the parietal and temporal lobes has shown to be involved in inhibitory control of Go/No-Go and Stop tasks [22]. Additionally, bilateral superior, inferior, and dorsolateral prefrontal cortices; supplementary motor area; anterior cingulate gyrus; inferior parietal and temporal lobes; caudate nucleus and cerebellum have been found to be activated in Go/No-Go tasks [24, 29, 32, 36, 37]. The bilateral inferior parietal lobes are thought to be involved in cognitive switching as tested in Switch-Task [3, 16, 34], as also our results confirm.

Neuroimaging studies of response inhibition have identified a set of regions, which are activated in conditions that require withholding a motor response with functional abnormalities in frontal striato-thalamic and fronto-parieto-cerebellar regions [18, 27, 28, 35]. In Stop task, participants are required to perform in a choice reaction time task with infrequent Stop-trials embedded [31]: the Go-signal is followed after a short delay by the Stop-signal indicating to withhold the response. This requires the cancellation of an already prepared response. The Go/No-Go paradigm is a selective attention task with a relatively low load on inhibitory control. Specifically, a network comprising the inferior frontal gyrus (IFG), dorsolateral PFC, inferior parietal lobule, pre-supplementary motor area (pre-SMA) and basal ganglia nuclei is typically found in fMRI-studies of response inhibition [2, 9, 26]. Our results are in line with the existing literature.

Task switching is an executive function and tests cognitive flexibility that involves the ability to shift attention between one task to another. This ability allows a person to rapidly and efficiently adapt to different situations by inhibiting one response and executing an alternative one [7, 21]. Inferior frontal-striatal networks are thought to mediate the inhibitory process underlying both motor response inhibition and cognitive switching [4, 11]. This is also supported by our data.

ADHD subjects were comparable to controls in the speed of inhibiting a response and did equally correct the task performance compared to controls on fMRI Go/No-Go task. In the sub-group analysis, ADHD+ group showed generally decreased activation, whereas ADHD− group showed generally increased activation at Go/No-Go task. Also at the Switch task, ADHD− group showed hyperactivation compared to the two other groups, controls and ADHD+, respectively.

The Stop signal paradigm [14] is a specific tool to measure inhibitory control. The Stop task measures the ability to withhold “last minute” already triggered motor response. A motor response that has already been triggered by predominant Go-signals has to be withheld when the signal is unpredictably, infrequently, and relatively quickly followed by a stop signal. In the literature, a Stop task has been used to show reduced activation in inferior prefrontal cortex, caudate and anterior cingulate [10, 39]. However, this task did not show a clear pattern to distinguish the three groups robustly from each other in our study.

Interestingly, behavioral parameters didn't vary statistically significantly between the three groups. However, Go/No-Go task together with Switch task produced the best contrast between the groups: medication-naïve ADHD subjects are showing hyperactivation compared to the ADHD+ and control groups, respectively. FMRI might add additional information to the brain activation pattern differences and possible treatment response of adults with ADHD. Information concerning the morphological substrate of ADHD might prove to be a toll that allows to monitor and quantify the effect of the medical treatment.

### Limitations

Block designs have been criticized in the cognitive test literature [2, 29, 37]. However, studies using event-related designs have not been much more accurate in the observed activation foci, even though event-related designs are more specific in correlating brain activation to the inhibitory targets [12]. Further limitation of our study is relatively small size of the sub-groups and that the results are not corrected for multiplicity. Although there is diagnostic criteria for the diagnosis of ADHD, there is a spectrum of differential diagnosis and some overlapping features of such differential diagnosis with ADHD that needs to be considered when making the diagnosis of ADHD. FMRI patterns in patients with border line personality, bipolar disorder and substance abuse [34] to name a few may also show similarities in fMRI that need criteria for distinguishing them from the ADHD patients. Also, no data were collected about the patient`s movement patterns which might have highlighted further characteristics between the studied groups. The behavioral pattern of ADHD patients has been described with increasingly impaired attention, such as difficulties in focusing and in performing tasks.

## Conclusion

FMRI studies of motor task inhibition and cognitive switching—two important features in ADHD− demonstrate the potential to differentiate adults with ADHD and without ADHD, as well patients with ADHD under and without stimulant medication. FMRI opens possibly a new window for monitoring the therapeutic effect of ADHD medication. Further prospective validation studies are needed.

## Data Availability

On a request to the corresponding author.

## Abbreviations

ACC: Anterior cingulate cortex
ADHD−: Adult attention deficit hyperactivity disorder
ADHD: Medication-naïve adults with the diagnosis of ADHD
ADHD+: Medicated adults with the diagnosis of ADHD
BOLD: Blood oxygen level dependent
DTVP-A: Developmental test of visual perception–adolescent and adult
EPI: Echo planar imaging sequence
FDR: False discovery rate
fMRI: Functional MRI
FWHM: Full width half maximum
IFG: Inferior frontal gyrus
PFC: Prefrontal cortex
Pre-SMA: Pre-supplementary motor area
RFX: Random-effect analysis
T: Tesla
ta: Acquisition time
TE: Echo time
TI: Inversion time
TR: Repetition time
tse: Turbo spin echo
y: Years
